# Welcome note INIC12 organizing committee

**DOI:** 10.5334/ijic.1041

**Published:** 2012-09-04

**Authors:** Paolo Pasini

**Affiliations:** Instituta per la Sicurezza Sociale San Marino

With great satisfaction I would like to warmly welcome our colleagues participating in this conference. I would probably seem haughty if I started by saying that San Marino is a small country with a big health care system, however, nobody can deny that the organizational and operational structure of San Marino health care system is really oriented towards great aims.

If we consider the peculiarity of joining together health care, social care and social security into a single institution, this can somehow anticipate the concept of integration.

The constant attention to the territory connected to and directed by the same organizational structure makes our health care system a tested prototype of integrated health care.

The most important challenge of this system is to take care of more and more complex needs in view of absolutely non-homogeneous professionalism and organizations.

“No man is an island”, recites a poem by John Donne.

No specialised discipline and no professional alone will be able to answer to the demand for health care from citizens of the twentieth century.

The organization will be able to accept the challenge by ensuring integration of disciplines, sources and operators.

Integration goes through awareness that harmonic growth of our body, mutual knowledge and osmosis among the parts is able to react adequately to the changed health needs.

Equity and solidarity principles as a basis of the San Marino health system have also been deep-rooted among the population of this small community and perhaps you can feel a natural tendency to an integrated care atmosphere.

So I hope that you will face this conference as an open window of a real experiment on integrated care.

I hope that the work we will carry out can offer you many innovative ideas and can give a great contribution to the study of integrated care systems.

Thank you, enjoy your stay, and I wish you well in your work.

